# Macrophage A2aR Alleviates LPS‐Induced Vascular Endothelial Injury and Inflammation via Inhibiting M1 Polarisation and Oxidative Stress

**DOI:** 10.1111/jcmm.70458

**Published:** 2025-03-05

**Authors:** Yanxiu Li, Tingzhen Chen, Iokfai Cheang, Peiben Liu, Lin Zhao, Xiaoxin He, Yuxi Jin, Mingmin Tang, Zhongqi Zhang, Chengyu Sheng, Zhongwen Zhang, Xiangrong Zuo

**Affiliations:** ^1^ Department of Critical Care Medicine The First Affiliated Hospital of Nanjing Medical University Nanjing China; ^2^ State Key Laboratory for Innovation and Transformation of Luobing Theory, Department of Cardiology The First Affiliated Hospital of Nanjing Medical University Nanjing China; ^3^ Department of Critical Care Medicine The Second Hospital of Nanjing Nanjing China; ^4^ Jiangsu Province Key Laboratory of Neurodegeneration Nanjing Medical University Nanjing China; ^5^ Department of General Surgery The Affiliated Jiangning Hospital of Nanjing Medical University Nanjing China

**Keywords:** adenosine A2a receptor, endotoxemia, macrophage M1 polarisation, oxidative stress, vascular endothelial injury

## Abstract

Vascular inflammation and endothelial dysfunction secondary to unchecked activation of endothelium are key mechanisms underlying sepsis and organ failure. However, the intrinsic processes that mitigate excessive endothelial cell activation remain incompletely understood. To determine the central role of adenosine A2a receptor (A2aR) on macrophages in modulating lipopolysaccharide (LPS)‐induced vascular endothelial dysfunction, we constructed macrophage A2aR‐conditional knockout (Mac‐A2aR KO) mice, and stimulated the mice and macrophages with LPS. A2aR agonist, CGS21680, was administered to these models to further explore its impact. Results showed that knockout of Macrophage A2aR exacerbated LPS‐induced vascular permeability, oedema, inflammatory cardiac damage and upregulated expression of intercellular adhesion molecule‐1 (ICAM‐1) and E‐selectin in cardiopulmonary vascular endothelium. Moreover, deletion of A2aR on macrophages also markedly aggravated LPS‐induced increases in reactive oxygen species (ROS) and declines in antioxidant enzyme gene mRNA and protein expression levels related to oxidative stress (OS). Furthermore, deficiency of A2aR in bone marrow‐derived macrophages (BMDMs) promotes LPS‐induced macrophage M1 polarisation and secretion of inflammatory cytokines, especially tumour necrosis factor‐alpha (TNF‐α). Conversely, the pretreatment with CGS21680 in vivo and in vitro showed corresponding improvement in functions of vascular endothelial dysfunction. These data demonstrate that A2aR in macrophages represents a promising novel therapeutic target for LPS‐induced uncontrolled vascular endothelial injury and inflammation potentially through reducing macrophage M1 polarisation and OS and inhibiting the production and release of TNF‐α production.

## 1 Introduction

Sepsis is a severe, life‐threatening condition resulting from a dysregulated host response to infection, leading to multiorgan dysfunction syndrome (MODS) and associated with high mortality [1]. One of the primary mechanisms of sepsis is widespread vascular leakage and inflammation due to endothelial activation and dysfunction [2]. This activation induces the upregulation of adhesion molecules essential for the recruitment of polymorphonuclear neutrophils (PMNs) and macrophages [3, 4]. For instance, E‐selectin mediates their rolling, while intercellular adhesion molecule‐1 (ICAM‐1) mediates their adhesion [5, 6]. Additionally, another study has highlighted that inhibiting excessive endothelial activation can protect against the inflammatory response [7]. However, the underlying mechanisms and specific pharmacological therapies for mitigating excessive activation of vascular endothelial cells remain unclear [8].

Extracellular adenosine binds to adenosine receptors (A1R, A2aR, A2bR and A3R), with A2aR predominantly expressed in immune cells, particularly macrophages, regulating inflammatory processes [9, 10]. Activation of A2aR exhibits anti‐inflammatory, tissue‐protective and immunosuppressive effects [11, 12, 13], suggesting the therapeutic potential in treating various inflammatory diseases and ischaemia–reperfusion injury [14, 15, 16, 17]. The above studies suggest that A2aR may be closely related to cardiovascular endothelial injuries. However, the role and mechanism of A2aR in endothelial activation remain unclear.

The free‐radical theory of sepsis posits that excessive oxidative stress (OS) induces proinflammation, impaired vasoreactivity and endothelial hyperpermeability, leading to MODS [18, 19]. The excess reactive oxygen species (ROS) exacerbate such oxidative damage and chronic inflammation [20]. Consequently, it is hypothesised that activating macrophage A2aR may mitigate endothelial damage, emphasising the specific molecular mechanisms of cell damage induced by OS.

Macrophages are the major producers of ROS during sepsis, contributing to oxidative damage [21]. Lipopolysaccharide (LPS) exposure induces ROS generation in macrophages, which mimics the pathophysiology of sepsis [22]. The complex interendothelial junctional structures of endothelial cells make their interaction with macrophages crucial in inflammatory diseases, angiogenesis, vascular inflammation and sepsis [23, 24, 25, 26].

Given that macrophage mechanisms for vascular endothelial injury and the participation of ROS in LPS‐induced sepsis [27], we hypothesised that LPS‐induced vascular inflammation could also be alleviated by activation of macrophage A2aR via reduction of OS. Using LPS‐induced macrophage A2aR‐conditional knockout (Mac‐A2aR KO) mice, our study aims to validate the protective role of A2aR against endothelial damage exacerbated by increased OS and M1 polarisation of macrophages.

## 2 Materials and Methods

### 2.1 Mouse Models of Vascular Inflammation and Animal Procedures

C57BL/6J mice were obtained from the Animal Core Facility of Nanjing Medical University. Mac‐A2aR KO mice were generated by intercrossing Lyz2‐cre mice with A2aR^f/+^ mice (The Jackson Laboratory, Cat #:010678) as previously described [28], resulting in Lyz2‐Cre; A2aR^f/f^ mice.

Vascular inflammation was induced in male Mac‐A2aR KO mice and wild‐type (WT) mice (10–12 weeks old) by intraperitoneal injection of LPS (5 mg/kg, Beyotime, China), with the control group receiving sterile saline [29]. Male and female Mac‐A2aR KO mice and C57BL/6J mice (8 weeks old) were injected intraperitoneally with LPS (5 mg/kg) combined with CGS21680 hydrochloride (1 mg/kg, MCE, USA) or DMSO (Sigma, USA) for 24 h (h). Serum and target organs were collected at the specified time points indicated.

### 2.2 Qualitative and Quantitative Determination of Organ Edema and Vascular Permeability

Evans blue dye at a dose of 30 mg/kg was injected into the caudal vein of mice 1 h before tissue collection. Tissues were perfused with cold phosphate‐buffered saline (PBS), blotted dry, weighed and snap‐frozen in liquid nitrogen. The tissues were then homogenised in PBS for 4 min and incubated with 1.0 mL N,N‐dimethylformamide (MACKLIN, China) for 24 h at 56°C, followed by centrifuging at 10,000 g for 20 min. The absorbance of lung, liver and kidney supernatants was determined at 620 nm. The extravasated Evans blue‐conjugated albumin (EBA) concentration in lung, liver and kidney homogenates was calculated against a standard curve.

Vascular permeability was evaluated by near‐infrared fluorescence (NIRF) imaging [30, 31]. Following sacrifice and fixation in 4% paraformaldehyde (PFA), hearts, lungs, livers and kidneys were dissected and then imaged using the IVIS Spectrum imaging system (Perkier, Waltham, MA, USA). Fluor 540 and CFSE‐related fluorescence signals were separated from the auto‐fluorescence signals using Living Image software (PerkinElmer). Organ oedema was assessed by measuring the tissue dry‐to‐wet (W/D) weight ratio, and vascular permeability was measured using tissue EBA flux [32, 33].

### 2.3 Procedures of Macrophages and Human Umbilical Vein Endothelial Cells (HUVECs)

Macrophages were treated with DMSO (control), CGS21680 (10 nM), LPS (1 μg/mL) or LPS + CGS21680 (pretreated with 100 nM CGS21680 for 30 min before 12 h LPS exposure). The supernatants were collected as conditioned media (CM) and used to culture HUVECs.

PMA‐differentiated THP‐1 cells were polarised towards the M1 or M2 phenotype by incubation for 48 h with IFN‐γ (20 ng/mL, PeproTech, USA) and LPS (100 ng/mL) or with IL‐4 (20 ng/mL, PeproTech) and IL‐13 (20 ng/mL, PeproTech), respectively [34].

OS in PMA‐differentiated THP‐1 cells was induced or inhibited by a 12 h treatment with H_2_O_2_ (200 μM) or N‐acetyl‐L‐cysteine (NAC, cytosolic ROS scavenger, 20 mM, Beyotime), respectively.

### 2.4 Quantitative Real‐Time Polymerase Chain Reaction (qRT‐PCR)

Total RNA was isolated using RNAiso Plus (TAKARA, Japan), followed by reverse transcription with a cDNA reverse transcription kit (EZB or Vazyme, China); qRT‐PCR analysis was performed using SYBR Green qPCR Master Mix (EZB or Vazyme). The primers used for analysis are listed in Table S1. Data were presented as the fold change relative to control.

### 2.5 Intracellular ROS Production Assays

PMA‐differentiated THP‐1 cells were treated as indicated, followed by incubation with DCFH‐DA (10 μM, Beyotime) at 37°C for 20 min in a dark environment. The cells were then washed with medium without FBS three times, digested by digestion solution (0.25%) and then centrifuged at 1000 rpm for 5 min. The 10% supernatant was transferred and fluorescence at 488/525 nm was measured. The 90% supernatant was lysed with RIPA buffer containing protease inhibitor cocktail and PMSF. All the ROS data were normalised with protein concentration calculated by the BCA Protein Assay Kit.

### 2.6 Flow Cytometry

Mouse heart and lung tissue were suspended in PBS buffer and incubated with allophycocyanin‐conjugated anti‐mouse F4/80 (Biolegend, USA), fluorescein isothiocyanate‐conjugated anti‐mouse CD45 (Biolegend), phycoerythrin anthocyanin 7‐conjugated anti‐mouse CD206 (Biolegend), Brilliant Violet 650‐conjugated anti‐mouse CD11b (Biolegend), Brilliant Violet 510‐conjugated anti‐mouse CD11c (Biolegend) and the matching control isotype IgGs for 30 min at 4°C. The cells were washed and resuspended in PBS buffer, and analysed by flow cytometry (NovoCyte Flow cytometry). M1 macrophages were identified as F4/80^+^ and CD11c^+^, and M2 macrophages were identified as F4/80^+^ and CD206^+^. Data analysis was performed using NovoExpress software.

### 2.7 Statistics Analyses

One‐way ANOVA or two‐way ANOVA was used to test differences for multiple groups. Student's t‐test was performed for pairwise comparisons. Power calculations were used to determine appropriate sample sizes for all in vivo studies. Data were expressed as mean ± standard error of mean (SEM), and differences were considered significant when *p* < 0.05.

## 3 Results

### 3.1 LPS Treatment Reduces A2aR Expression and Causes the Generation of Oxidative Stress

**FIGURE 1:**
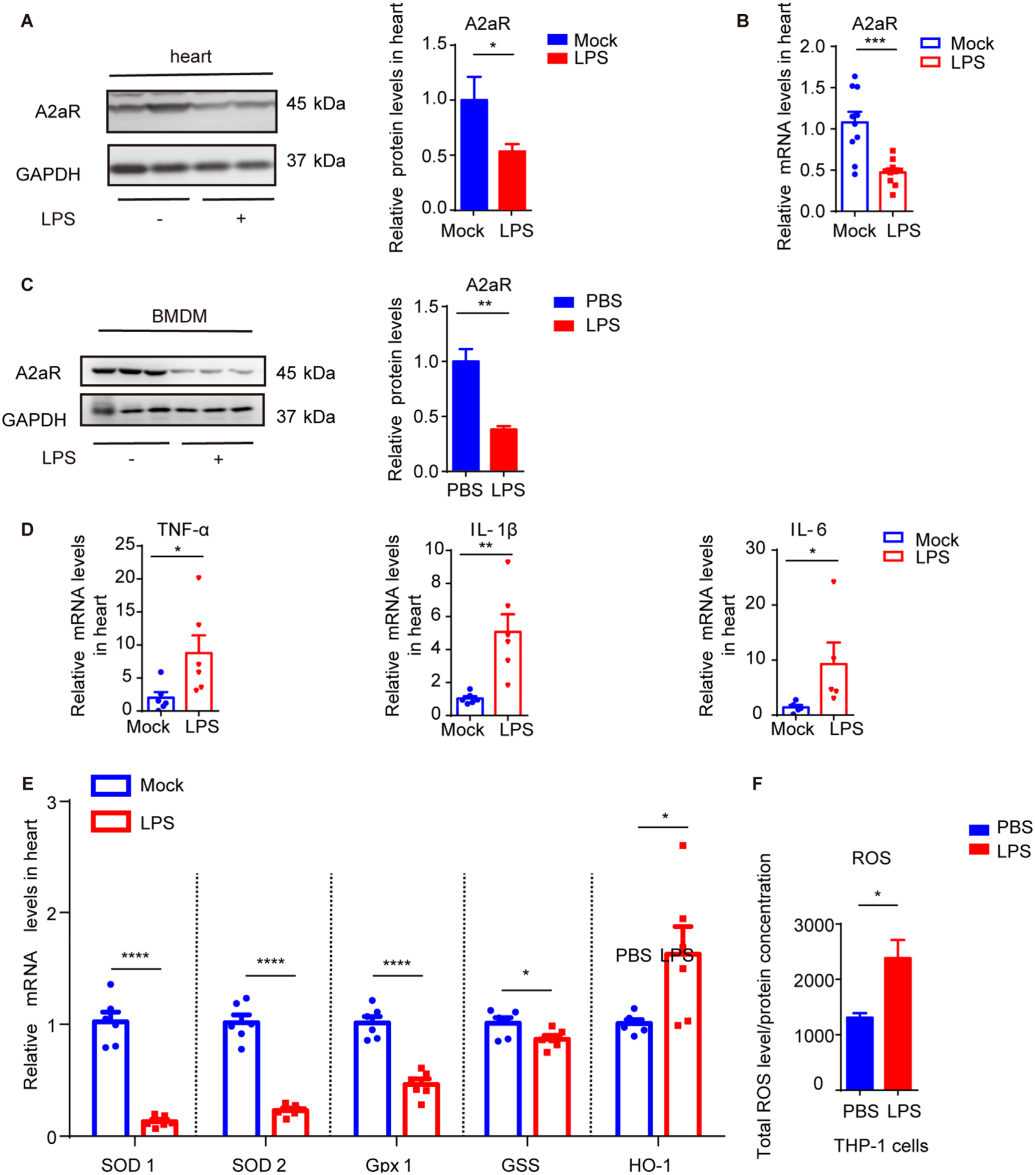
LPS treatment reduces A2aR expression and causes the generation of oxidative stress. (A) Representative western blot (WB) images and quantification of A2aR protein expression levels in hearts of mice injected with LPS (5 mg/kg) or 0.9% normal saline (NS) for 24 h^.^ (*n* = 5). (B) Quantitative PCR (qRT‐PCR) of A2aR mRNA expression levels (*n* = 10). (C) A2aR protein expression levels in primary bone marrow‐derived macrophages (BMDMs) after treatment with PBS or LPS (1 μg/mL, 12 h) (*n* = 3). (D) QRT‐PCR of tumour necrosis factor‐alpha (TNF‐α), IL‐1β and IL‐6 mRNA expression levels in mice hearts injected with LPS (*n* = 5–6). (E) QRT‐PCR of superoxide dismutase Type 1/2 (SOD1/2), glutathione peroxidase 1 (Gpx1) and heme oxygenase 1 (HO‐1) mRNA expression levels in mice hearts injected with LPS (5 mg/kg) or 0.9% NS for 24 h (*n* = 6). (F) Measurement of total reactive oxygen species (ROS) from PMA‐differentiated THP‐1 cells challenged with LPS (1 μg/mL, 12 h). ROS values normalised to protein concentration (*n* = 3). Error bars represent mean ± SEM. *p or #*p* < 0.05, **p or ##*p* < 0.01.

To investigate the relationship between LPS‐induced inflammatory response and A2aR expression, we first analysed A2aR protein and mRNA levels in LPS‐treated mice hearts using western blot (WB) and qRT‐PCR. Following LPS treatment, A2aR protein and mRNA expression levels were reduced by approximately 50% (Figure 1A,B). Similarly, primary BMDMs challenged with LPS exhibited a greater than 60% decrease in A2aR protein levels compared to controls (Figure 1C). LPS also induced significantly higher mRNA expression levels of TNF‐α, IL‐1β and IL‐6 in the mice hearts (Figure 1D). Moreover, LPS treatment reduced mRNA expression of enzymatic antioxidants including SOD1, SOD2, Gpx1 and GSS, while increasing HO‐1 and ROS levels (Figure 1E,F).

### 3.2 Knockout Macrophage A2aR Enhanced Endotoxemia‐Induced Oxidative Stress, Vascular Permeability and Oedema

**FIGURE 2:**
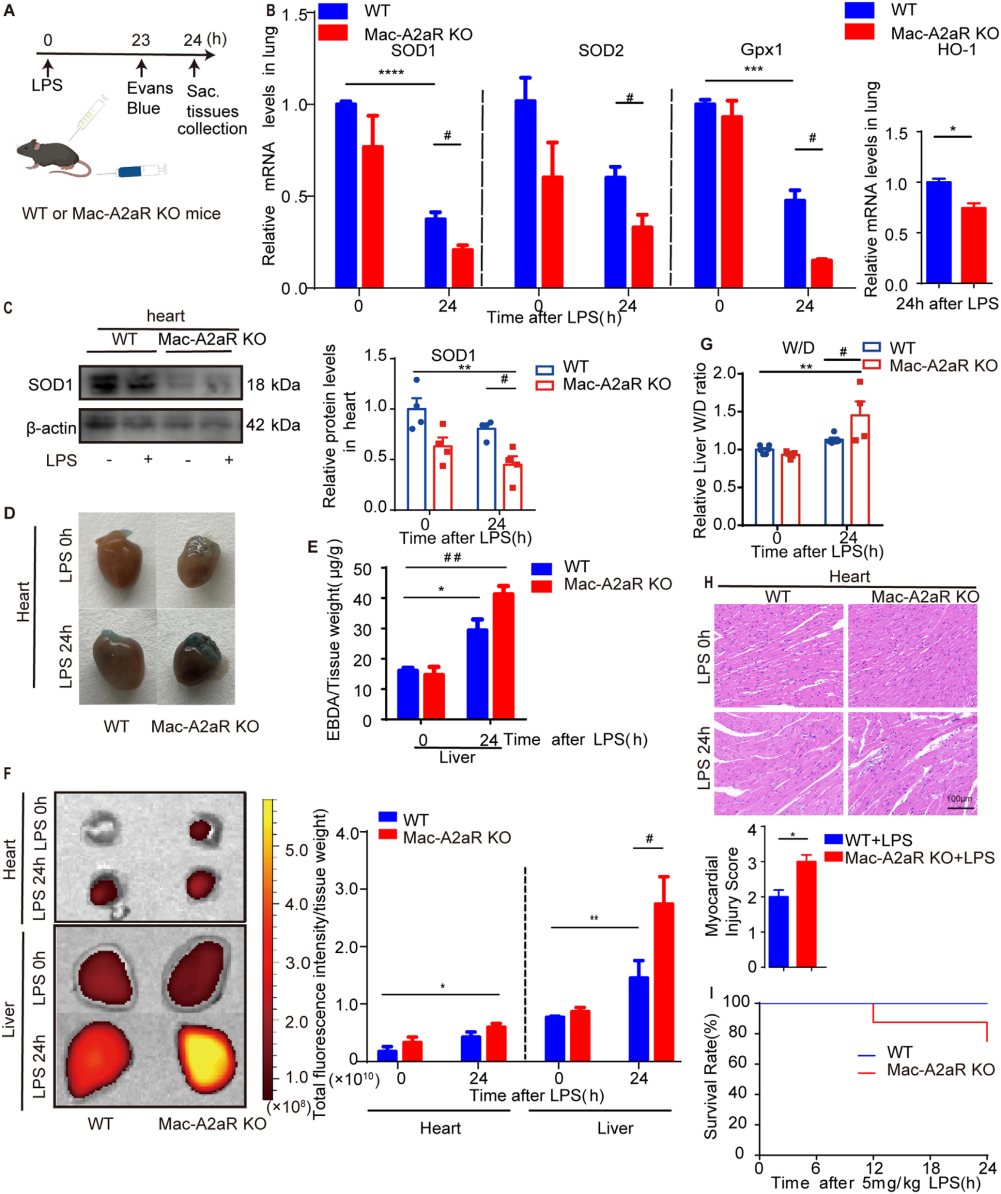
Knockout macrophage A2aR enhanced endotoxemia‐induced oxidative stress, vascular permeability and oedema. (A) Schematic illustration showed that WT and Mac‐A2aR KO mice were challenged with LPS (5 mg/kg, i.p., 24 h) or vehicle. Evans Blue was injected (i.v.) 1 h before the tissue collection. (B) The mRNA expression of SOD1, SOD2, Gpx1 and HO‐1 in lung tissues from the above groups (*n* = 3–4). (C) WB analysis of SOD1 protein expression levels in the heart (*n* = 3–4). (D and E) Vascular permeability of heart and liver measured by Evans blue‐conjugated albumin (EBA) extravasation. (D) Representative heart appearance after EBA administration. (E) Quantitative analysis of EBA extravasation in the liver (*n* = 2–3). (F) Intravascular near‐infrared fluorescence imaging of the heart and liver, with total fluorescence intensity calculation. (G) Dry‐to‐wet (W/D) weight ratio of liver (*n* = 4–6). (H) Heart histology. Magnification 40 ×, scale bar: 100 μm. Myocardial injury score (*n* = 3). (I) 24 h survival rates after LPS treatment (*n* = 8), Mantel–Cox log‐rank test. Error bars represent mean ± SEM. *p or #*p* < 0.05, **p or ##*p* < 0.01, ***p or ###*p* < 0.001, ****p or ####*p* < 0.0001.

In the Mac‐A2aR KO mice, the protein levels of A2aR were significantly lower compared to WT mice (Figure S1A,B). Upon LPS challenge, SOD1, SOD2 and Gpx1 mRNA expression, as well as SOD1 protein expression, were further downregulated in Mac‐A2aR KO mice compared to WT (Figure 2B,C). Mac‐A2aR KO mice demonstrated exacerbated oedema in the heart and liver, with increased vascular hyperpermeability as indicated by enhanced EBA extravasation (Figure 2D,E) and NIRF imaging (Figure 2F), respectively. Moreover, Mac‐A2aR KO mice showed significantly higher W/D in the liver after LPS challenge compared to the WT mice (Figure 2G). Notably, histological analysis revealed severe cardiac abnormalities in LPS‐induced Mac‐A2aR KO mice compared to WT mice, including disrupted cardiomyocyte arrangement, widened myocardial fibre septa and myocardial fibre breakage (Figure 2H). However, there was no significant difference in the 24‐h survival curve(Figure 2I).

### 3.3 Knockout Macrophage A2aR Aggregated Endotoxemia‐Induced Cardiopulmonary Vascular Endothelial Adhesion and Injury In Vivo

**FIGURE 3:**
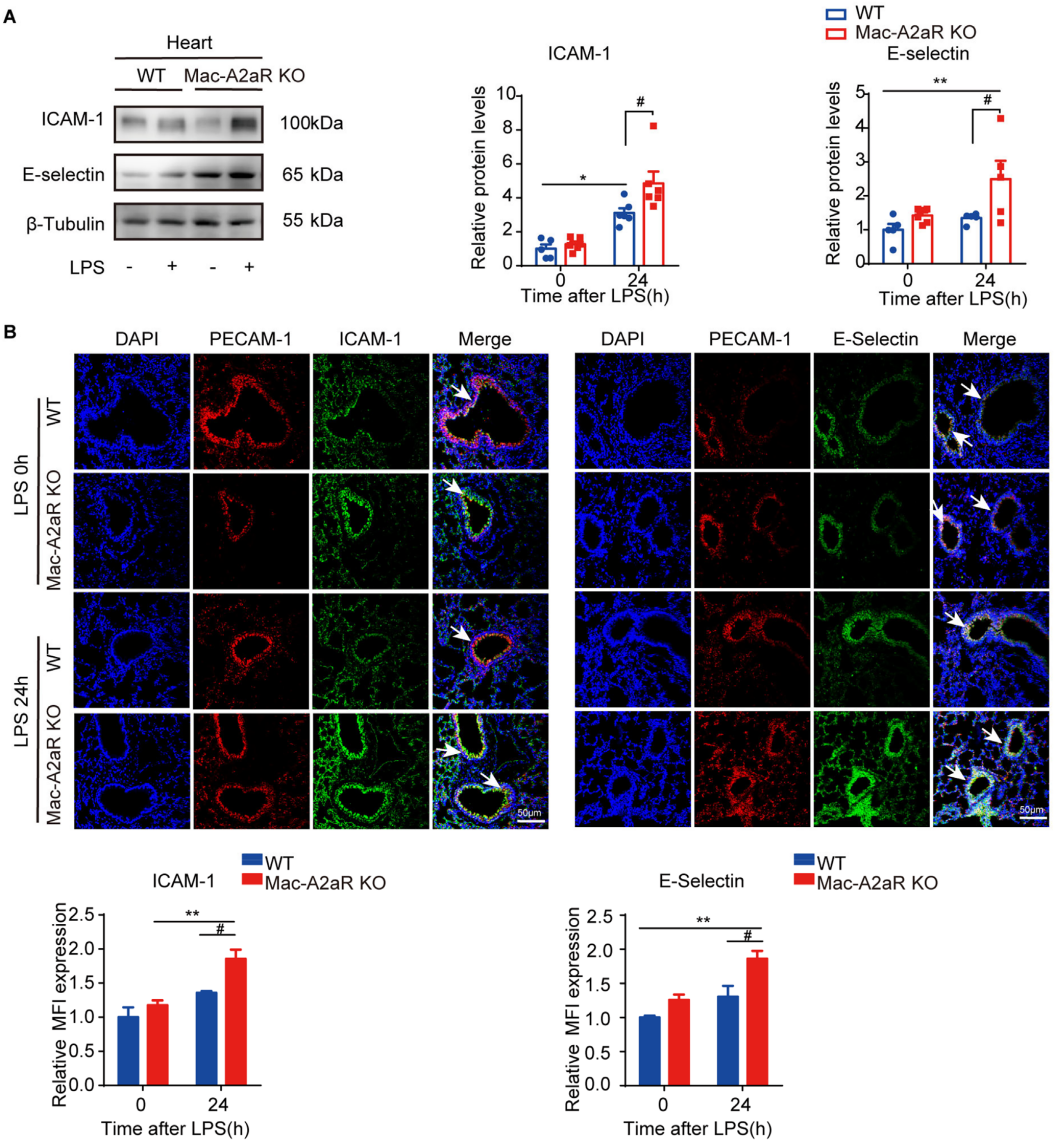
Knockout macrophage A2aR aggregated endotoxemia‐induced cardiopulmonary vascular endothelial adhesion and injury in vivo. (A) Representative WB images and quantification of intercellular adhesion molecule‐1 (ICAM‐1) and E‐selectin protein expression levels in heart from WT or Mac‐A2aR‐KO mice after LPS (5 mg/kg, i.p.,24 h) challenge (*n* = 5–6). (B) Immunofluorescence images of E‐selectin and ICAM‐1 stained with platelet endothelial cell adhesion molecule‐1 (PECAM‐1) in lungs by immunofluorescence staining. Scale bar: 50 μm. Quantitative data illustrated the changes in relative mean fluorescent intensity (MFI) of ICAM‐1 and E‐selectin expression. Error bars represent mean ± SEM. *p or #*p* < 0.05, **p or ##*p* < 0.01.

We further assessed the effects of macrophage A2aR on endothelial adhesion by evaluating endothelial adhesion markers. We found significantly higher expression of E‐selectin and ICAM‐1 protein in the hearts of LPS‐treated Mac‐A2aR KO mice compared to WT mice (Figure 3A). Immunofluorescence staining confirmed colocalisation of PECAM‐1 with ICAM‐1 and E‐selectin in pulmonary endothelial cells (Figure 3B).

### 3.4 Activation of A2aR Attenuates LPS‐Induced Oxidative Stress and Vascular Endothelial Dysfunction In Vivo

**FIGURE 4:**
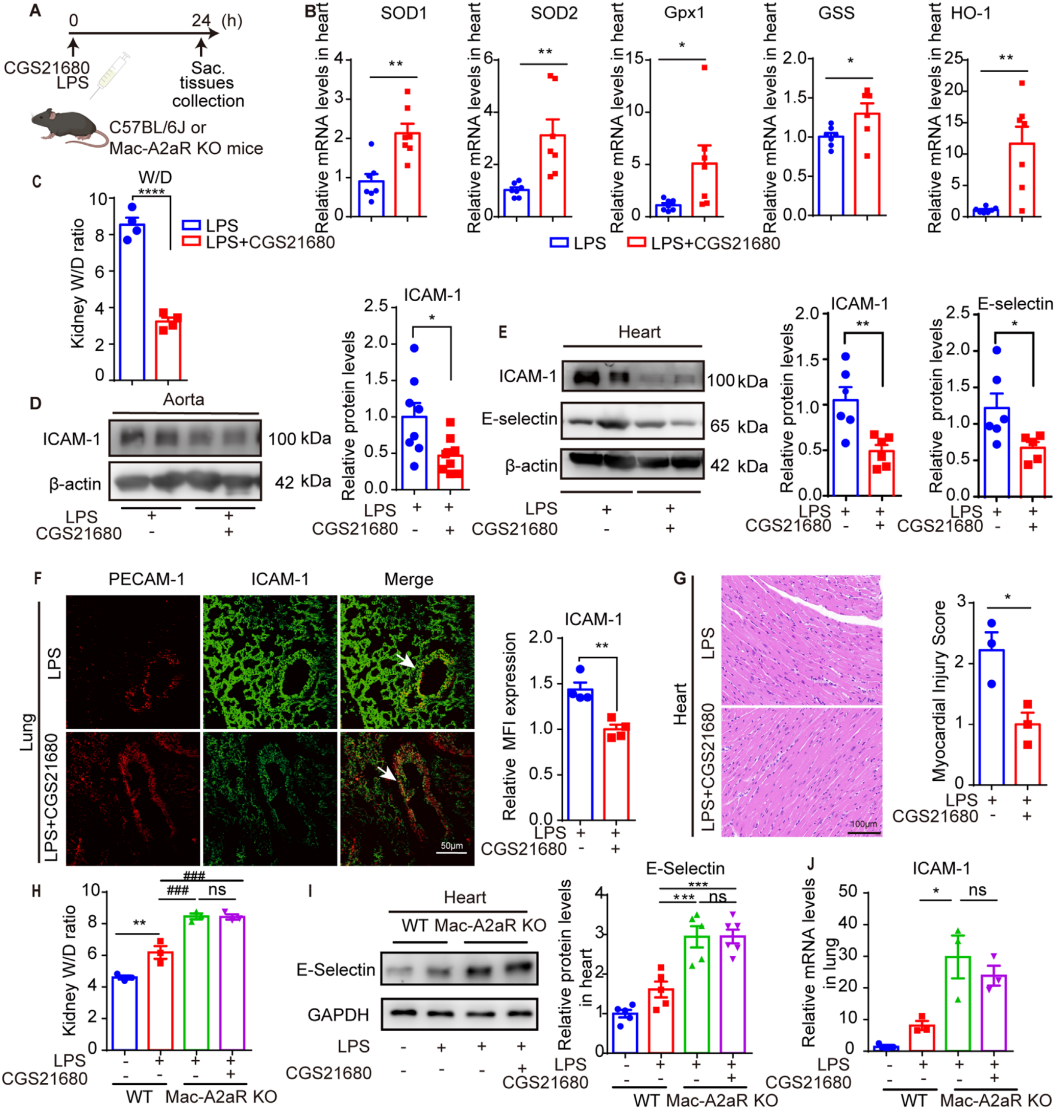
Activation of A2aR attenuates LPS‐induced oxidative stress and vascular endothelial dysfunction in vivo. (A) Schematic illustration showed that CGS21680 (1 mg/kg) or dimethyl sulfoxide (DMSO) was instilled combined with LPS (5 mg/kg) challenge for 24 h. (B) SOD1, SOD2, Gpx1 and HO‐1 mRNA expression levels in the heart (*n* = 6–7). (C) The kidney W/D ratio was measured as W/D (*n* = 4). (D) Representative WB images and quantitation of ICAM‐1 protein expression in the aorta (*n* = 8). (E) Representative WB images and quantitation of ICAM‐1 and E‐selectin protein expression in the heart (*n* = 6). (F) Representative Immunofluorescence images and quantitation of relative ICAM‐1 MFI in pulmonary vascular endothelial marked by PECAM‐1 (*n* = 4). Scale bar: 50 μm. (G) Heart histology. Myocardial injury score (*n* = 3). Magnification 40 ×. (H) WT and Mac‐A2aR‐KO mice were treated with LPS (5 mg/kg) and CGS21680 (1 mg/kg) or PBS for 24 h. Assessment of kidney oedema (*n* = 3). (I) WB of E‐selectin protein expression levels in the heart (*n* = 5). (J) QRT‐PCR of ICAM‐1 mRNA expression levels in the lungs. *p or #*p* < 0.05, **p or ##*p* < 0.01, ***p or ###*p* < 0.001.

Treatment with CGS21680 significantly restored expression levels of OS‐related genes (SOD1, SOD2, Gpx1 and GSS) and increased HO‐1 expression in LPS‐induced mice (Figure 4B). CGS21680 treatment also reduced kidney W/D ratio, indicating improved oedema (Figure 4C). Furthermore, CGS21680 treatment reduced E‐selectin and ICAM‐1 protein expression in the aorta, heart and lung vessels in LPS‐challenged mice (Figure 4D–F), and mitigated the corresponding heart inflammatory damage (Figure 4G).

Conversely, in Mac‐A2aR KO mice, the protein expression levels of A2aR were similar between the groups pretreated with or without CGS21680 (Figure S2A,B). CGS21680 pretreatment failed to alleviate LPS‐induced tissue oedema or reduce E‐selectin and ICAM‐1 expression levels in heart and lung tissues (Figure 4H,I, Figure S2C). These results suggest that activation of A2aR by CGS21680 in normal cells can mitigate LPS‐induced tissue oedema, heart damage and endothelial activation. However, for macrophages from Mac‐A2aR KO mice, CGS21680 failed to provide these protective effects.

### 3.5 Disruption of Macrophages A2aR Aggregates LPS‐Induced Enhanced Oxidative Stress and Macrophages M1 Polarisation

**FIGURE 5:**
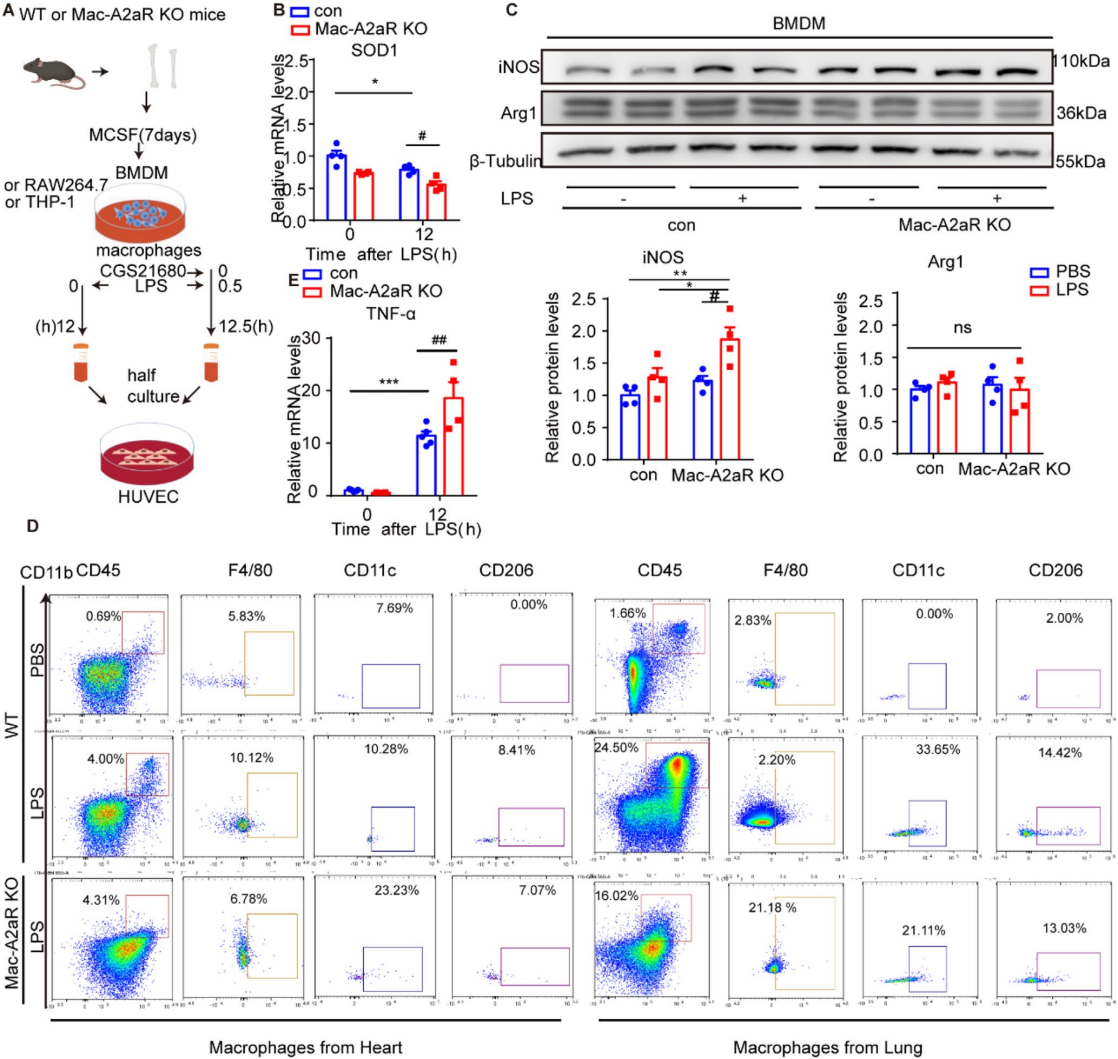
Disruption of macrophages A2aR aggregates LPS‐induced enhanced oxidative stress and macrophages M1 polarisation. (A) Schematics of BMDMs obtained from Mac‐A2aR‐KO mice or WT mice stimulated by LPS (1 μg/mL) for 12 h. (B) SOD1 mRNA expression levels (*n* = 4). (C) Representative images and quantification of inducible nitric oxide synthase (iNOS) and arginase 1 (Arg1) protein expression levels (*n* = 4–5). (D) Macrophages sorted from heart and lung from Mac‐A2aR‐KO mice or WT mice after LPS (5 mg/kg, i.p.) or PBS treatment for 24 h. Flow cytometry analysis of macrophages in heart and lung (CD45 + CD11b + F4/80+), M1‐like macrophages (CD45 + CD11b + F4/80 + CD11c+) and M2‐ like macrophages (CD45 + CD11b + F4/80 + CD206+). (E) QRT‐PCR of TNF‐α mRNA expression levels in BMDMs (*n* = 4–5). *p or #*p* < 0.05, **p or ##*p* < 0.01, ***p or ###*p* < 0.001. ns represents no significant difference.

To investigate the mechanism of A2aR in regulating endothelial activation, BMDMs were isolated from Mac‐A2aR KO mice (Figure 5A). Following LPS challenge, mRNA expression of SOD1 was significantly decreased in control mice and was further reduced in Mac‐A2aR KO mice (Figure 5B). Protein levels of inducible nitric oxide synthase (iNOS, M1 marker) were elevated with A2aR downregulation, whereas arginase 1 (Arg1 and M2 marker) remained unaffected (Figure 5C). Through flow cytometry, heart and lung macrophages from LPS‐treated mice confirmed enhanced M1 polarisation in Mac‐A2aR KO mice compared to WT control (Figure 5D). The production of pro‐inflammatory cytokines in the systemic inflammatory response was then examined [35]. The result revealed that A2aR deletion significantly exacerbated LPS‐induced increased mRNA expression of TNF‐α (Figure 5E).

### 3.6 Activation of Macrophage A2aR Attenuates LPS‐Induced Increased Oxidative Stress, M1 Polarisation and Production of TNF‐α

**FIGURE 6:**
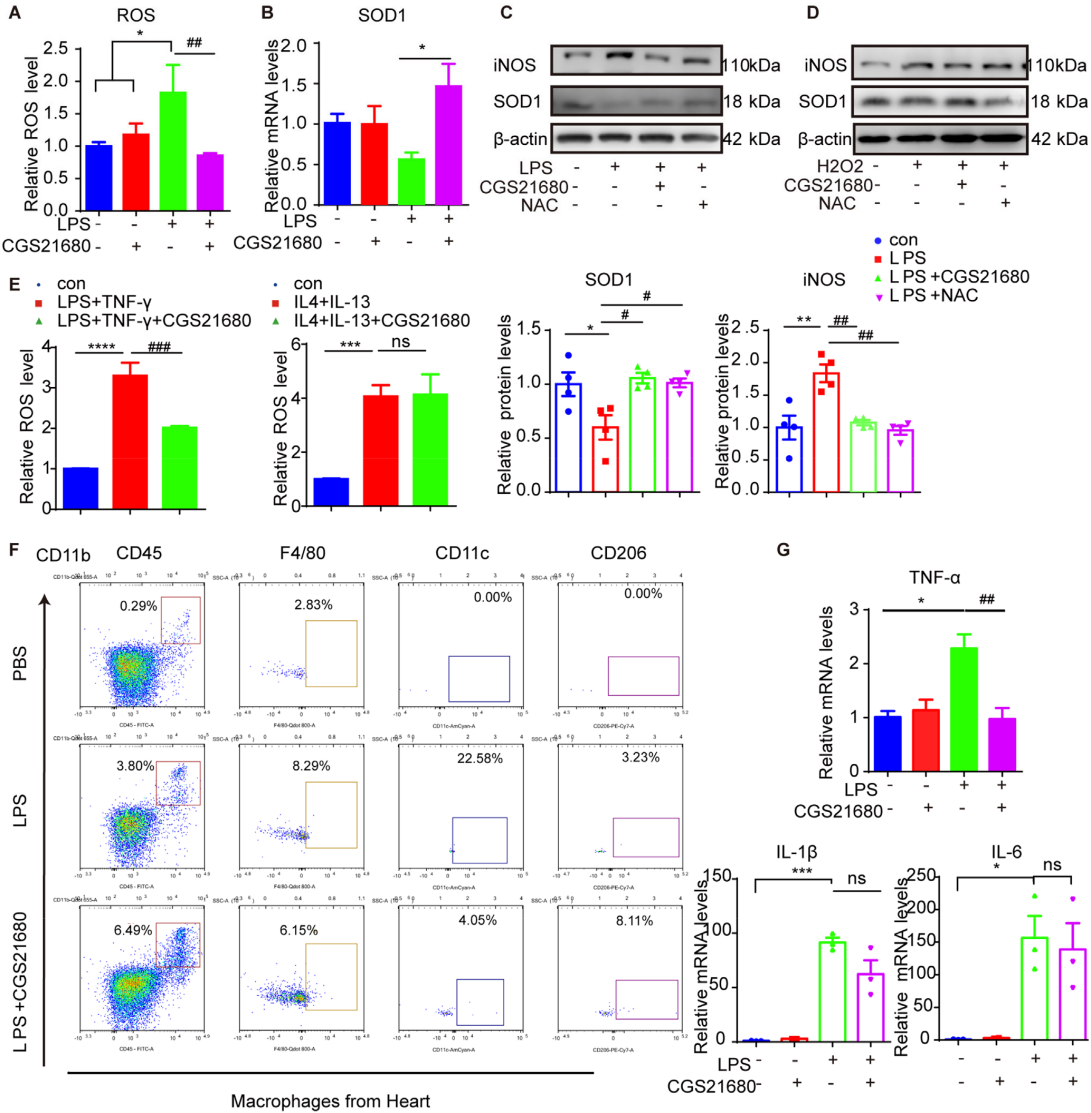
Activation of macrophage A2aR attenuates LPS‐induced oxidative stress and M1 polarisation, reducing TNF‐α production. (A) Intracellular total ROS was detected in PMA‐differentiated THP‐1 cells treated with vehicle, CGS21680 (10 nM), LPS (1 μg/mL) or LPS after 30 min CGS21680 pretreatment (*n* = 3). (B) QRT‐ PCR of SOD1 mRNA expression levels in BMDMs processed as (A) above (*n* = 3–4). (C and D) PMA‐differentiated THP‐1 cells were treated with vehicle, LPS (1 μg/mL), LPS after 30 min CGS21680 pretreatment (10 nM) or LPS after 30 min N‐acetyl‐L‐cysteine (NAC) pretreatment (20 mM); H_2_O_2_ (200 μM), H_2_O_2_ after 30 min CGS21680 pretreatment (10 nM) and H_2_O_2_ after 30 min NAC pretreatment (20 mM). WB of iNOS and/or SOD1 protein levels expression in cell groups (*n* = 4). (E) Intracellular total ROS was detected in PMA‐differentiated THP‐1 cells treated with vehicle, LPS (100 μg/mL) and interferon‐gamma (IFN‐γ) (20 ng/mL) with or without 30 min CGS21680 pretreatment before; interleukin (IL)‐4 (20 ng/mL) and IL‐13 (20 ng/mL) with or without 30 min CGS21680 pretreatment before (*n* = 3). (F) Flow cytometry analysis of macrophages sorted from WT mice heart after LPS (5 mg/kg, i.p.) with CGS21680(1 mg/kg) or DMSO treatment for 24 h. (G) The mRNA expression levels of TNF‐α, IL‐1β and IL‐6 from the BMDMs treated as (A) above (*n* = 3). *p or #*p* < 0.05, **p or ##*p* < 0.01, ***p or ###*p* < 0.001 and ****p or ####*p* < 0.0001.

We then treated LPS‐challenged macrophages with CGS21680 and observed that 30 min pretreatment with CGS21680 significantly suppressed ROS generation and restored SOD1 mRNA levels in LPS‐challenged macrophages (Figure 6A,B). We further evaluated the effects of NAC (a ROS scavenger) or CGS21680 on LPS or H_2_O_2_‐induced OS in differentiated THP‐1 cells. Consistent with NAC pretreatment, CGS21680 pretreatment suppressed the reduction of SOD1 and the increase of iNOS protein expression caused by LPS (Figure 6C,D).

Specifically, CGS21680 mitigated ROS levels in M1‐polarised macrophages (Figure 6E). Flow cytometry analysis also showed that CGS21680 treatment reduced LPS‐induced M1 polarisation in heart macrophages (Figure 6F). Finally, we assessed the expression of pro‐inflammatory cytokines and found that pretreatment with CGS21680 significantly decreased the LPS‐induced upregulation of TNF‐α mRNA expression, while showing a slight reduction in the LPS‐induced increase of IL‐6 or IL‐1β mRNA expression in BMDMs (Figure 6G).

### 3.7 Macrophage A2aR Regulates Endothelial Cell Overactivation

**FIGURE 7:**
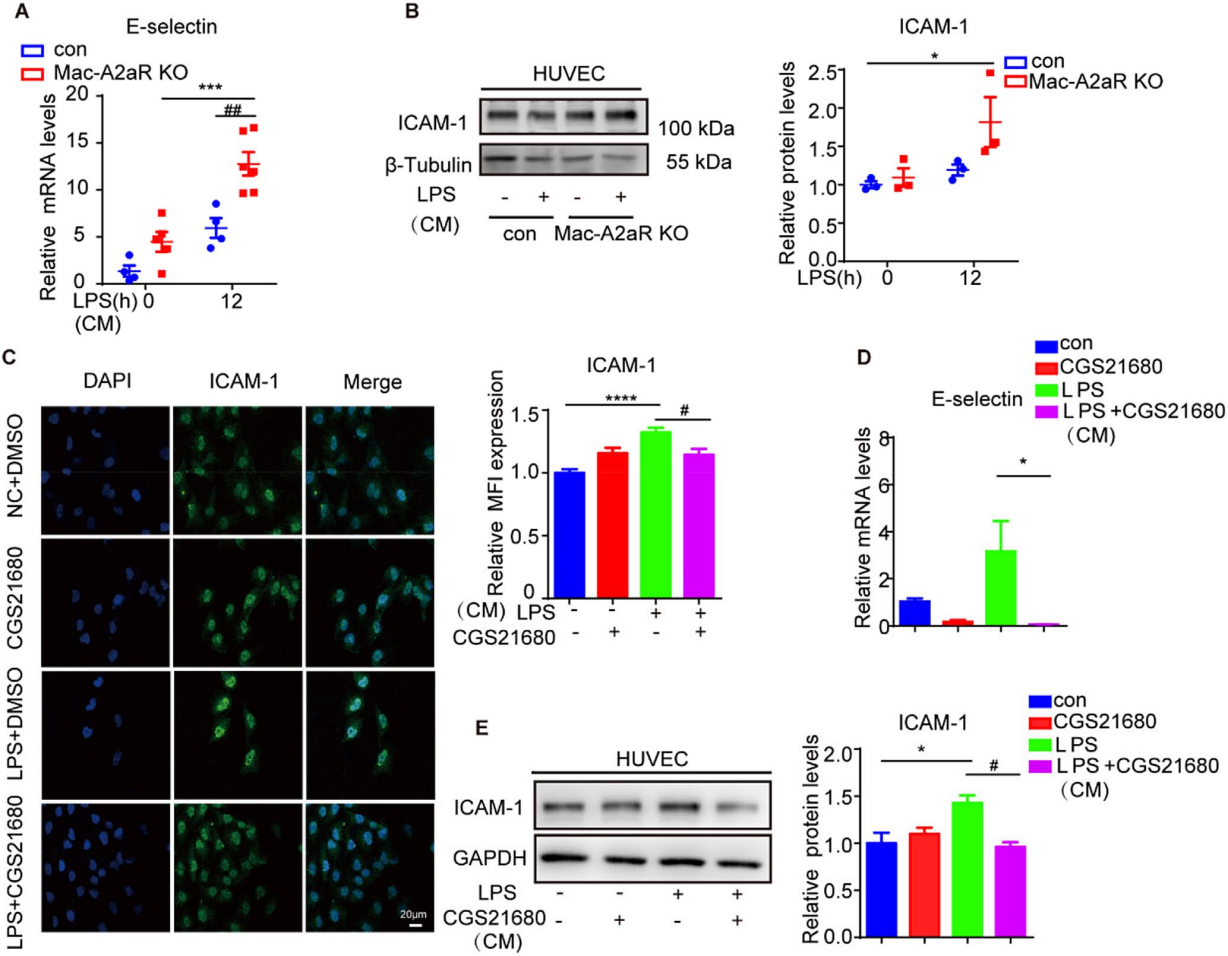
Macrophage A2aR regulates endothelial cell overactivation. (A) QRT‐PCR of E‐selectin mRNA expression levels in human umbilical vein endothelial cells (HUVEC) cultured with conditioned medium (CM) of BMDMs from Mac‐A2aR‐KO mice or WT mice (*n* = 4–6). (B) WB and quantitative analysis of ICAM‐1 protein expression levels in HUVECs (*n* = 3). (C) Immunofluorescence staining for ICAM‐1 in HUVECs cultured with CM from RAW264.7 cells. Scale bar: 20 μm. (D) E‐selectin mRNA in HUVECs cultured with CM from PMA‐differentiated THP‐1 cells (*n* = 3). (E) WB and quantitative analysis of ICAM‐1 protein expression levels in HUVECs cultured with CM from RAW264.7 cells (*n* = 3). *p or #*p* < 0.05, **p or ##*p* < 0.01, ***p or ###*p* < 0.001 and ****p or ####*p* < 0.0001.

To investigate whether A2aR activation enhances vascular endothelial repair, macrophages from WT or Mac‐A2aR KO mice were treated with LPS and CGS21680 or DMSO, and their conditioned media were used to culture HUVECs, as illustrated in Figure 5A. Conditioned media from BMDMs of LPS‐challenged Mac‐A2aR KO mice increased E‐selectin mRNA levels in HUVECs by approximately 2.5 times higher compared to media from BMDMs of LPS‐challenged WT mice (Figure 7A). Furthermore, ICAM‐1 protein expression was similarly elevated (Figure 7B).

Conversely, qRT‐PCR analysis revealed that E‐selectin mRNA expression in HUVECs cultured with media from macrophages treated with both CGS21680 and LPS was reduced by 98% compared to those cultured with media from macrophages treated with LPS alone (Figure 7D). Similar results were observed in the changes in ICAM‐1 protein expression, as detected by immunofluorescence (Figure 7C) and WB (Figure 7E).

## 4 Discussion

In this study, we observed significantly decreased A2aR protein expression levels in the hearts and macrophages of LPS‐treated mice, underscoring its crucial role in the inflammatory response. Knockout of macrophage A2aR markedly enhanced OS and M1 polarisation in LPS‐treated mice or macrophages, increased the production and release of the pro‐inflammatory cytokine TNF‐α, which consequently led to excessive endothelial activation, elevated the release of adhesion molecules ICAM‐1 and E‐selectin and heightened vascular permeability. This sequence of events exacerbated tissue oedema, vascular endothelial permeability and cardiopulmonary injury. Conversely, activation of macrophage A2aR inhibited LPS‐induced OS, macrophage M1 polarisation and release of TNF‐α, thereby alleviating vascular endothelial injury and inflammation.

Macrophages A2aR, activated by adenosine, play a pivotal role in modulating inflammatory responses and immune function by increasing their sensitivity to the anti‐inflammatory effect of adenosine [36]. Macrophages are among the most critical leukocytes in the pathogenesis of endothelial injury during sepsis. Studies have shown that LPS downregulates the overall protein expression level of A2aR in the lung tissues of endotoxemia mice, and A2aR knockout aggravates LPS‐induced vascular damage in the liver and lungs [13, 37]. However, Murphree et al. found that LPS treatment significantly increases the mRNA expression level of macrophage A2aR [38], which contrasts with our finding that endotoxemia downregulates the protein expression level of macrophage A2aR. This expression variability may result from differences in experimental conditions, macrophage types and LPS treatment concentrations.

Macrophages are among the most critical leukocytes in the pathogenesis of endothelial injury during sepsis. Besides, A2aR is broadly expressed in various immune cells, including T cells, monocytes, dendritic cells and natural killer cells, where it plays anti‐inflammatory and immunosuppressive roles. However, current research on A2aR in T cells and dendritic cells predominantly focuses on tumours and autoimmune diseases [39].

Sepsis‐induced OS is a key factor in vascular endothelial damage. OS impairs cells and tissues, causing increased permeability, proadhesion and impaired vasoreactivity [18]. Studies have demonstrated that vascular endothelial injury can be ameliorated by downregulating protein kinase C zeta‐mediated OS and apoptosis [40]. In addition, antioxidant enzymes have been reported to attenuate the formation of lipid peroxides and mitigate oxidative processes, preserving the equilibrium between OS and the antioxidative defence mechanism [41, 42]. SOD catalyses the dismutation of the superoxide radical into ordinary molecular oxygen, while GPX protects the biofilms from ROS damage and upholds cellular functionality [43]. In our study, LPS‐induced mice exhibited upregulated mRNA expression of HO‐1. However, the knockout of macrophage A2aR significantly reduced HO‐1 mRNA expression after LPS treatment. Conversely, activation of macrophage A2aR promoted HO‐1 upregulation in LPS‐treated mice. This may be due to the partial collapse of the antioxidant system during sepsis, leading to the upregulation of specific antioxidants like HO‐1 to combat OS. As inflammation progresses, the antioxidant system fully collapses, resulting in a decline in overall antioxidant enzymes [44, 45]. In conclusion, activation of macrophage A2aR reduced the levels of OS, while the knockout of macrophage A2aR markedly enhanced LPS‐induced OS.

Besides, macrophages can exhibit diverse phenotypes through polarisation in response to various stimuli, thereby playing a critical role in modulating inflammation, facilitating tissue repair and maintaining homeostatic equilibrium. Suppression of ROS in RAW264.7 cells can diminish macrophage M1 polarisation during ischaemia–reperfusion injury [27]. Our analysis also showed that LPS‐ or H_2_O_2_‐induced OS promoted macrophage M1 polarisation, which could be diminished by CGS21680 or NAC. Deficiency of macrophage A2aR exacerbated LPS‐induced M1 polarisation. Moreover, LPS + IFN‐γ‐induced macrophage M1 polarisation amplified the increase of ROS production, which could be diminished by CGS21680. This suggested a mutual positive correlation between macrophage M1 polarisation and OS.

M1 macrophages release pro‐inflammatory mediators, while M2 macrophages release anti‐inflammatory mediators [46]. M1 polarisation leads to the overexpression of pro‐inflammatory factors such as TNF‐α, IL‐1 and IL‐6 [35]. Previous studies have highlighted that TNF‐α and IL‐1 induce endothelial activation, leading to increased vascular permeability, adhesion molecules and a procoagulant state [47, 48]. TNF‐α is recognised as one of the most extensively studied pro‐inflammatory cytokines [49]. Additionally, our observations suggest that the activation of macrophage A2aR can markedly inhibit LPS‐induced mRNA expression of TNF‐α, with a slight reduction in the mRNA expression of IL‐6 or IL‐1β, thereby lowering the expression levels of endothelial adhesion molecules ICAM‐1 and E‐selectin. However, the precise mechanisms underlying these effects remain unclear.

Our study has limitations. We could not exclude the potential effects of A2aR activation in other cell types when activating A2aR via CGS21680 in vivo. Moreover, LPS‐induced endotoxemia may not fully replicate the complexity of sepsis caused by live bacterial infections, potentially overlooking aspects of host–pathogen interactions and limiting generalisability. Future research should focus on specific macrophage subtypes and elucidate the precise mechanisms by which A2aR influences endothelial function.

## 5 Conclusions

In conclusion, our findings suggest that A2aR in macrophages represents a promising novel therapeutic target for LPS‐induced uncontrolled vascular endothelial injury and inflammation. A2aR may function by mitigating macrophage M1 polarisation and oxidative stress and inhibiting TNF‐α production and release, ultimately reducing endothelial overactivation and expression of ICAM‐1 and E‐selectin.

## Author Contributions

**Yanxiu Li:** validation (equal), visualization (equal), supervision (equal), project administration (equal), funding acquisition (equal). **Tingzhen Chen:** conceptualization (equal), methodology (equal), formal analysis (Supporting), writing – original draft preparation (equal), writing – review and editing (equal), visualization (equal). **Iokfai Cheang:** writing – original draft preparation (equal), writing – review and editing (equal), visualization (equal). **Peiben Liu:** methodology (equal). **Lin Zhao:** formal analysis (Lead). **Xiaoxin He:** software (equal), investigation (equal). **Yuxi Jin:** data curation (equal). **Mingmin Tang:** investigation (equal). **Zhongqi Zhang:** data curation (equal). **Chengyu Sheng:** resources (equal). **Zhongwen Zhang:** conceptualization (equal), software (equal), validation (equal), project administration (equal). **Xiangrong Zuo:** validation (equal), resources (equal), supervision (equal), project administration (equal), funding acquisition (equal). All authors have read and agreed to the published version of the manuscript.

## Ethics Statement

The animal study protocol was approved by the Institutional Review Board of the Institutional Animal Care and Use Committee of Nanjing Medical University (Protocol Code: IACUC‐2306010, Date of approval: 2023‐06‐05).

## Conflicts of Interest

The authors declare no conflicts of interest.

## Supporting information


Data S1.

Data S2.

## Data Availability

Data openly available in a public repository that issues datasets with DOIs.
