# Supplementary material for: Macrophage A2aR Alleviates LPS‐Induced Vascular Endothelial Injury and Inflammation via Inhibiting M1 Polarisation and Oxidative Stress
Source: J Cell Mol Med. 2025 Mar 5;29(5):e70458. doi: 10.1111/jcmm.70458 (PMC11882390; doi:10.1111/jcmm.70458)
Supplement: Supplementary file 1 — Data S1. [file JCMM-29-e70458-s002.pdf]

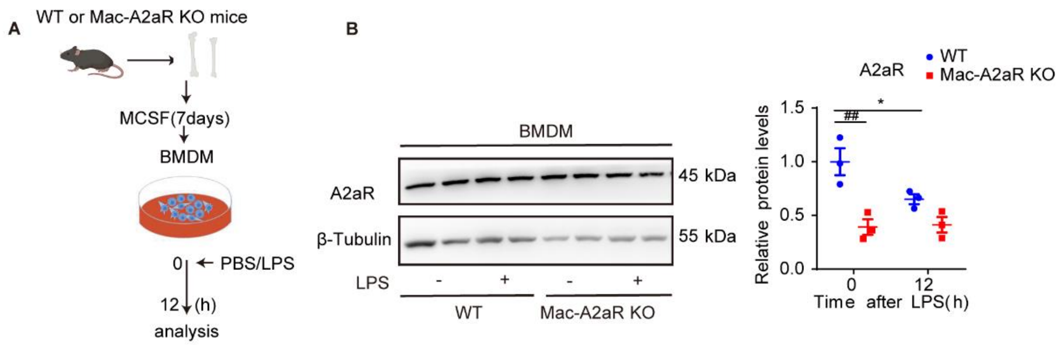

Supplementary Figure1. (A) BMDMs obtained from Mac-A2aR-KO mice or WT mice were stimulated by LPS (1 μg/mL) or PBS for 12h. (B) WB of A2aR protein expression levels in BMDMs. Quantitation of relative protein expression (n =3). Error bars represent mean ± SEM. *$p < 0.05$, ##$p < 0.01$.

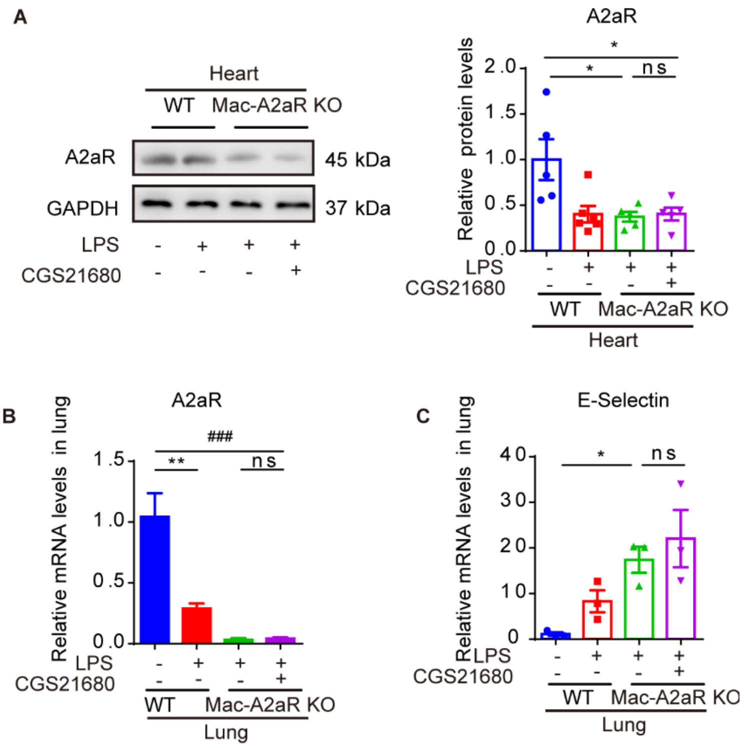

Supplementary Figure2. (A) WT and Mac-A2aR-KO mice were treated with LPS (5 mg/kg) and CGS21680 (1 mg/kg) or PBS for 24h. WB and quantitation of A2aR protein expression in the heart (n =5-6). (B) QPCR of A2aR mRNA expression levels in the lung tissues (n =3). (C) QPCR of E-selectin mRNA expression levels in the lung tissue (n =3).*p or #p <0.05, **p or ##p <0.01, ***p or ###p <0.001.
