# Supplementary material for: Macrophage A2aR Alleviates LPS‐Induced Vascular Endothelial Injury and Inflammation via Inhibiting M1 Polarisation and Oxidative Stress
Source: J Cell Mol Med. 2025 Mar 5;29(5):e70458. doi: 10.1111/jcmm.70458 (PMC11882390; doi:10.1111/jcmm.70458)
Supplement: Supplementary file 2 — Data S2. [file JCMM-29-e70458-s001.docx]

**Supplementary Methods**

*2.10 Western blotting (WB)*

Cells or heart and lung tissues were lysed in radioimmunoprecipitation assay (RIPA) buffer (Beyotime, #P0013B) supplemented with a protease inhibitor cocktail (Beyotime, #ST506) and phenylmethylsulfonyl fluoride (PMSF) (Roche, #0490683701). Lysates were sonicated and incubated for 30 min on the ice followed by centrifuging at 12,000 rpm for 15 min at 4°C. The protein concentration was determined with Enhanced BCA Protein Assay Kit (Beyotime, P0009). Equal amounts of samples were loaded for SDS-PAGE and transferred onto polyvinylidene fluoride (PVDF) membranes (Millipore, 0.2µm, ISEQ00010). The membranes were blocked with 5% nonfat milk and incubated with primary antibodies targeting A2aR (Immunoway, YT0129), ICAM-1 (ABclonal, A5597), E-selectin (Immunoway, YT5745), iNOS (Proteintech,18985-1-AP), Arg1 (Proteintech,16001-1-AP), or SOD1 (Proteintech,10269-1-AP) overnight at 4°C. Subsequently, the membranes were probed with HRP-conjugated secondary antibodies for 1h at room temperature. The protein bands were detected with High-sig ECL Substrate (Tanon, 180-5001). The relative band densities of the proteins were analyzed by using ImageJ Software.

*2.11 Immunofluorescence Staining*

For immunofluorescence staining, the frozen sections (15 µm) or deparaffinized and dehydrated sections (4 µm) from tissues or glass coverslips with cells fixed in 4% PFA solution were washed with PBS. Antigen retrieval was performed by microwave heating in citrate buffer for 15 min if necessary. The samples were treated with PBS containing 0.01% Triton X-100 for 10 min to facilitate permeabilization, followed by incubation with 5% BSA/PBS for 1 h to block the nonspecific antibody binding. After blocking, samples were incubated overnight at 4°C with primary antibodies, rinsed, and then incubated with appropriate fluorescently labeled secondary antibodies for 1-2 h at room temperature. To detect the adhesion of HUVECs, the lung tissue sections were double-stained for PECAM-1 (Proteintech, FITC-65058) and ICAM-1 (ABclonal, A5597), or PECAM-1 and E-selectin (Immunoway, YT5745). All sections were counterstained with nuclear 4′,6-diamidino-2-phenylindole (DAPI) for 7 min and mounted with an anti-fluorescence quench mounting medium. All fluorescence images were captured using an Olympus microscope and then analyzed with ImageJ.

*2.12 Hematoxylin and eosin staining (H&E)*

The heart was perfused with cold PBS to remove blood and then fixed with PFA for 24h. The tissues were dehydrated using graded ethanol and processed with paraffin. The tissues were then cut into 4µm thick sections and stained with modified HE stain kit (Solarbio, G1121).

*2.13 Survival Rates*

Following treatment of mice with LPS, the survival rates were determined every 12 h, 1 day. The survival rate is expressed as the percentage of live animals, and the Mantel-Cox log-rank test was used to determine differences between the experimental groups.

**Supplementary Table1.** **Primers for quantitative qRT-PCR.**

| Primer | Forward （5’-3’） | Reverse （5’-3’） |
| --- | --- | --- |
| Mouse：  A2aR  IL-1β  IL-6  TNFα  ICAM-1  E-Selectin  iNOS  Arg1  Sod1  Sod2  Gpx1  GSS  HO-1  β-actin  Human：  ICAM-1  E-Selectin  β-actin | CACGCAGAGTTCCATCTTCA  CTGGTACATCAGCACCTCAC  TGTATGAACAACGATGATGCACTT  AGTGACAAGCCTGTAGCCC  GTGATGCTCAGGTATCCATCCA  ATGCCTCGCGCTTTCTC  CAGATCGAGCCCTGGAAGAC  ACATTGGCTTGCGAGACGTA  AACCAGTTGTGTTGTCAGGAC  CAGACCTGCCTTACGACTATGG  AGTCCACCGTGTATGCCTTCT  CAAAGCAGGCCATAGACAGGG  CGCCTTCCTGCTCAACATT  GGCTGTATTCCCCTCCATCG  ATGCCCAGACATCTGTGTCC  TGTGGGTCTGGGTAGGAACC  CATGTACGTTGCTATCCAGGC | ATGGGTACCACGTCCTCAAA  AGAAACAGTCCAGCCCATAC  ACTCTGGCTTTGTCTTTCTTGTTATCT  GAGGTTGACTTTCTCCTGGTAT  CACAGTTCTCAAAGCACAGCG  GTAGTCCCGCTGACAGTATGC  CTGGTCCATGCAGACAACCT  ATCACCTTGCCAATCCCCAG  CCACCATGTTTCTTAGAGTGAGG  CTCGGTGGCGTTGAGATTGTT  GAGACGCGACATTCTCAATGA  AAAAGCGTGAATGGGGCATAC  TGTGTTCCTCTGTCAGCATCAC  CCAGTTGGTAACAATGCCATGT  GGGGTCTCTATGCCCAACAA  AGCTGTGTAGCATAGGGCAAG  CTCCTTAATGTCACGCACGAT |

*2.14 Preparation for macrophages and HUVECs*

Primary bone marrow-derived macrophages (BMDMs) from WT or Mac-A2aR KO mice were isolated by flushing the femur and tibia with either Dulbecco's modified Eagle's medium (DMEM) or RPMI1640 medium. Cell suspensions were then transferred to a new culture flask after 4 h and differentiated into macrophages through 7 days of stimulation with DMEM or RPMI1640 medium containing 10% fetal bovine serum (FBS, Gibco, 10099-141C) and 20 ng/mL macrophage colony-stimulating factor (MCSF, Peprotech, 315-02), or cultured with L929-conditioned complete RPMI1640 medium for a week to differentiate into macrophages. Murine RAW264.7 cell lines and phorbol 12-myristate 13-acetate (PMA, 200ng/mL, MCE, MY-18739)-stimulated THP-1 cell lines were cultured in complete RPMI1640 supplemented with 10% (vol/vol) FBS and 1% penicillin/streptomycin solution (P/S), while human umbilical vein endothelial cell (HUVEC) cell lines were cultured in endothelial cell medium containing 5% FBS (Sciencell, 0025), 1% endothelial cell growth factor (ECGS, Sciencell, 1052), and 1% P/S (Sciencell, 0503).
